# Slow points and adiabatic fixed points in recurrent neural networks

**DOI:** 10.1186/1471-2202-16-S1-P88

**Published:** 2015-12-18

**Authors:** Hendrik Wernecke, Claudius Gros

**Affiliations:** 1 Institute for Theoretical Physics, Goethe-University, Frankfurt am Main, 60438, Germany

## 

The time scales for cognitive information processing in the brain range, at least, from milliseconds (the time scale of the action potential) to many seconds (the time scale of short-term memory), spanning several orders of magnitude. In this context the slow dynamical components can be regarded as background processes modulating adiabatically the parameters governing the fast neural activity. For the case of recurrent neural networks the slow processes then change adiabatically the attractor landscape, including the adiabatic fixed points, inducing possibly both second order bifurcations, with respect to the steady-state neural activity, and first order catastrophes.

In this contribution we investigate the slow adaption of the attractor landscape of exemplary small recurrent neural networks consisting of continuous-time point neurons [1]. The state of one of these integrate-and-fire neurons is fully determined by its membrane potential and two adapting internal parameters [2], the gain and the threshold, with the time scale of adaption 1/ε being substantially larger than the time scale of the primary neural activity. We point out that not only the adiabatic fixed points of the network are important for shaping the neural dynamics, but also the points in phase space where the flow slows down considerably (called slow points or attractor ruins [3]).

**Figure 1:**
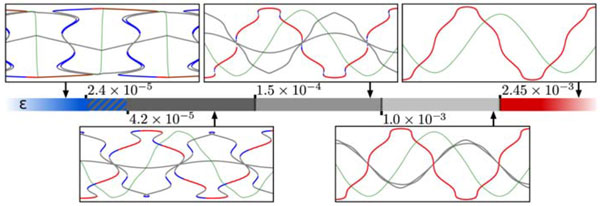
**Phase diagram of the three-neuron system showing five different phases distinguished by the stability and the shape of the adiabatic fixed points (AFP) for different values of the adaption rate ε**. One can observe three second order transitions (dashed) and one first order transition showing hysteresis (striped area). The subplots show the firing rate (green), saddle node AFP (gray), stable AFP (blue) and effectively attracting AFP (red) over time and represent a phase each.

We rigorously examine the metadynamics of the attractor landscape for a three-neuron system, observing five different phases (see Fig. 1) for different values of the adaption rate, of which four can be distinguished by the number and stability of the adiabatic fixed points. Three of the observed transitions are of second order. The remaining transition to the phase of lowest adaption is of first order and shows hysteresis. This transition occurs, remarkably, at very low adaption rates of ε ~ 10^-5 ^and is characterized by a higher-order catastrophe.

We conclude that even relatively simple recurrent networks may show highly non-trivial adapting attractor landscapes and that the study of the attractor metadynamics in the brain maybe important for a further understanding of decision processes and dynamical memory recall.
